# Supplementary material for: On the Effect of Vibrotactile Stimulation in Essential Tremor
Source: Healthcare (Basel). 2024 Feb 9;12(4):448. doi: 10.3390/healthcare12040448 (PMC10888095; doi:10.3390/healthcare12040448)
Supplement: Supplementary file 1 [file healthcare-12-00448-s001.zip › healthcare-2716600-supplementary.pdf]

Supplementary Materials: On the effect of vibrotactile stimulation in Essential Tremor

Ariana Moura Cabral [1*], Julio Salvador Lora-Millán [2], Adriano Alves Pereira [1], Eduardo Rocon [3] and Adriano de Oliveira Andrade [1]

Individualized Spectrum Analysis of Postural Tremor

Figures S1 to S15 provide an overview of the spectral analysis for the other 15 participants of the study. Each figure depicts the power spectral densities of the angular velocities of the hand and forearm for the participants during posture maintenance for both the trial without vibrotactile stimulation and the trial with vibrotactile stimulation. In addition, the figures highlight the spectral behaviour of the frequency bands adjacent to the peak frequency.

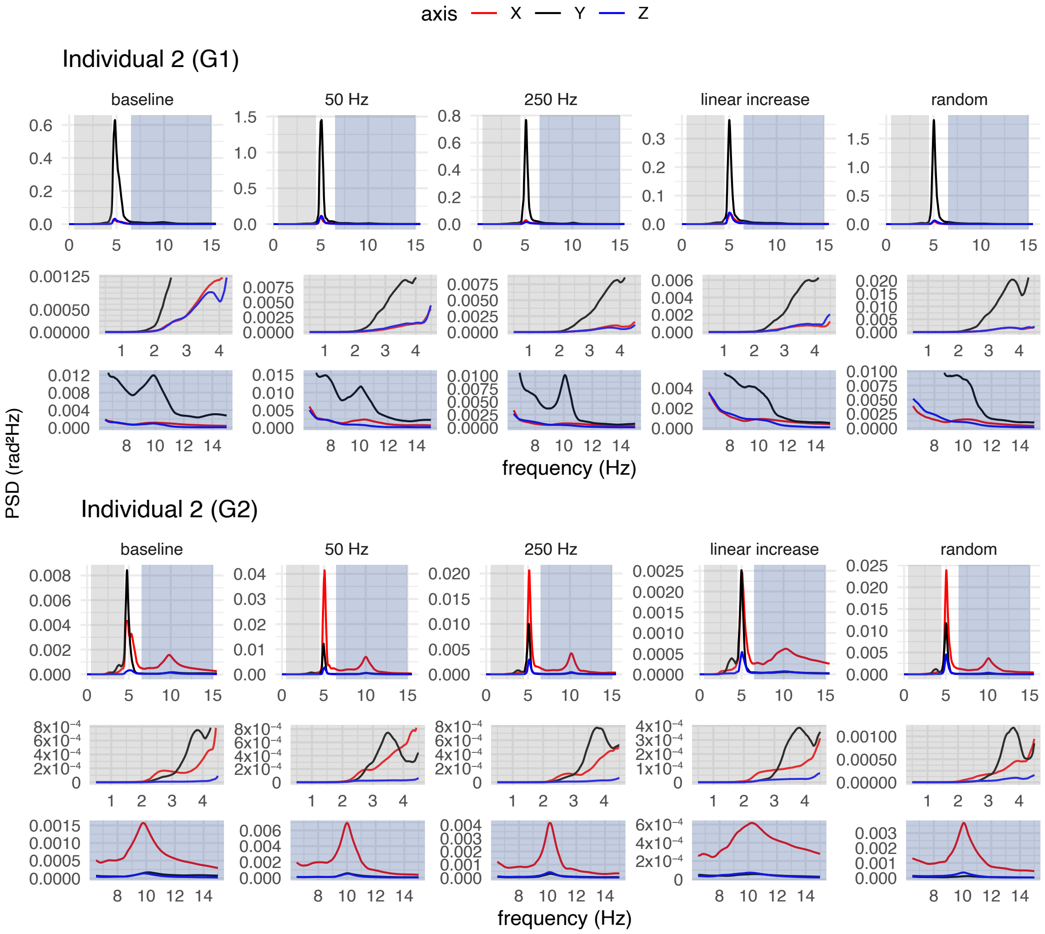

Figure S1. Power spectral densities of the angular velocity of the hand (G1) and forearm (G2) of **Individual 2** for each axis (X, Y and Z) during posture maintenance, without (baseline) and with (50 Hz, 250 Hz, linear and random increase) vibrotactile stimulation. The highlighted areas of the spectra (the gray and blue windows) depict the spectral behaviour for lower and higher frequency bands in relation to the peak frequency.

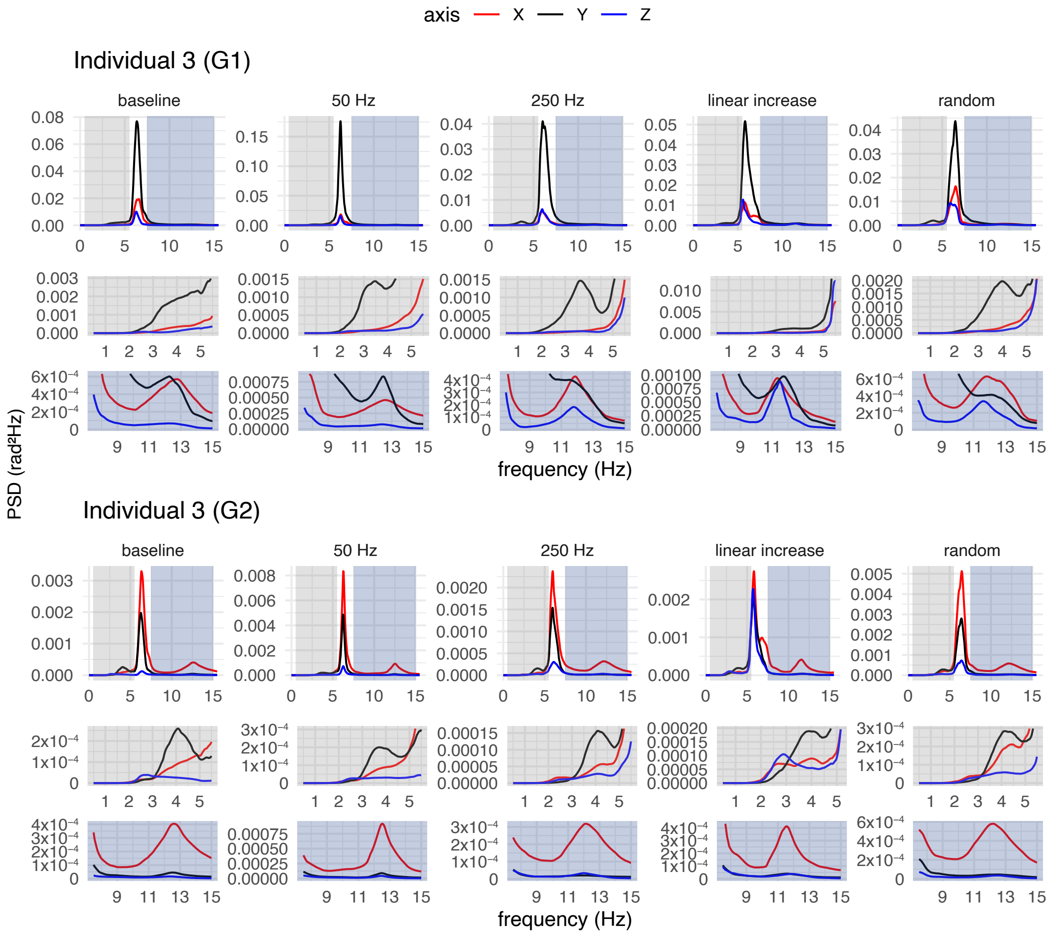

Figure S2. Power spectral densities of the angular velocity of the hand (G1) and forearm (G2) of **Individual 3** for each axis (X, Y and Z) during posture maintenance, without (baseline) and with (50 Hz, 250 Hz, linear and random increase) vibrotactile stimulation. The highlighted areas of the spectra (the gray and blue windows) depict the spectral behaviour for lower and higher frequency bands in relation to the peak frequency.

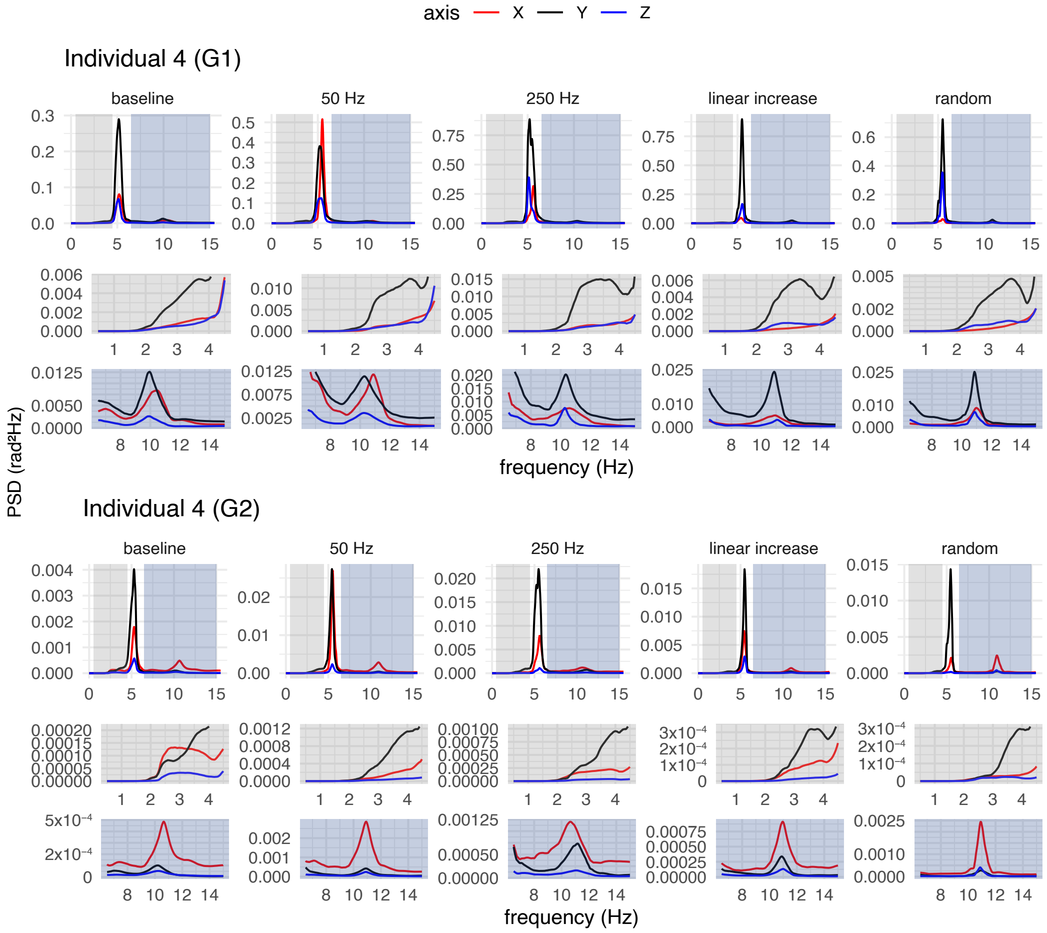

Figure S3. Power spectral densities of the angular velocity of the hand (G1) and forearm (G2) of **Individual 4** for each axis (X, Y and Z) during posture maintenance, without (baseline) and with (50 Hz, 250 Hz, linear and random increase) vibrotactile stimulation. The highlighted areas of the spectra (the gray and blue windows) depict the spectral behaviour for lower and higher frequency bands in relation to the peak frequency.

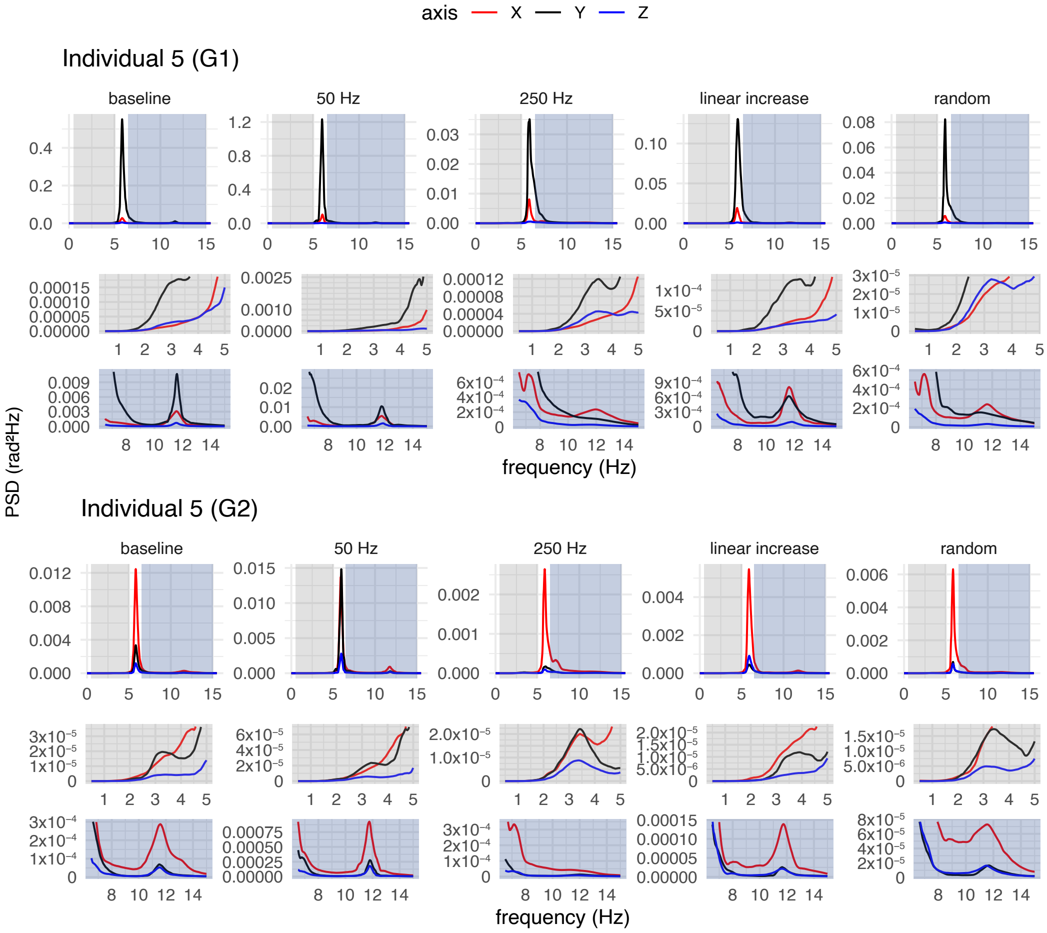

Figure S4. Power spectral densities of the angular velocity of the hand (G1) and forearm (G2) of **Individual 5** for each axis (X, Y and Z) during posture maintenance, without (baseline) and with (50 Hz, 250 Hz, linear and random increase) vibrotactile stimulation. The highlighted areas of the spectra (the gray and blue windows) depict the spectral behaviour for lower and higher frequency bands in relation to the peak frequency.

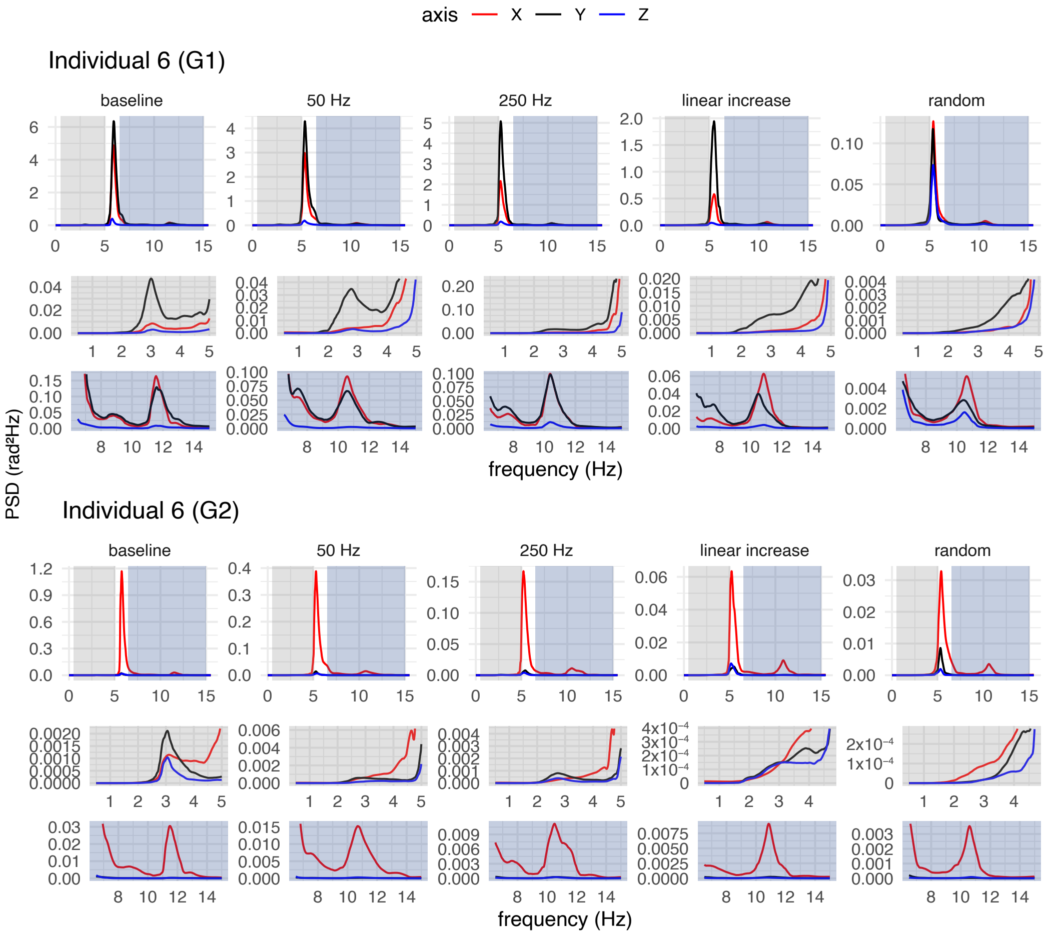

Figure S5. Power spectral densities of the angular velocity of the hand (G1) and forearm (G2) of **Individual 6** for each axis (X, Y and Z) during posture maintenance, without (baseline) and with (50 Hz, 250 Hz, linear and random increase) vibrotactile stimulation. The highlighted areas of the spectra (the gray and blue windows) depict the spectral behaviour for lower and higher frequency bands in relation to the peak frequency.

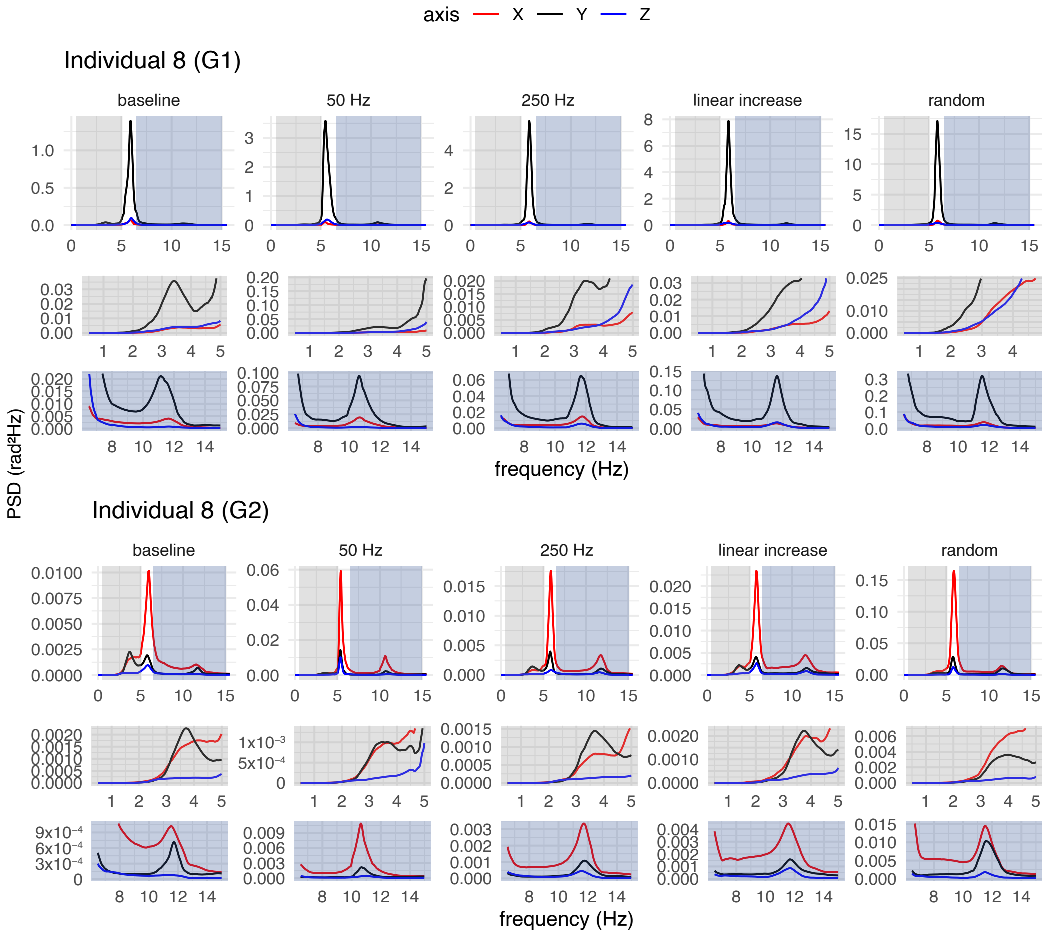

Figure S6. Power spectral densities of the angular velocity of the hand (G1) and forearm (G2) of **Individual 8** for each axis (X, Y and Z) during posture maintenance, without (baseline) and with (50 Hz, 250 Hz, linear and random increase) vibrotactile stimulation. The highlighted areas of the spectra (the gray and blue windows) depict the spectral behaviour for lower and higher frequency bands in relation to the peak frequency.

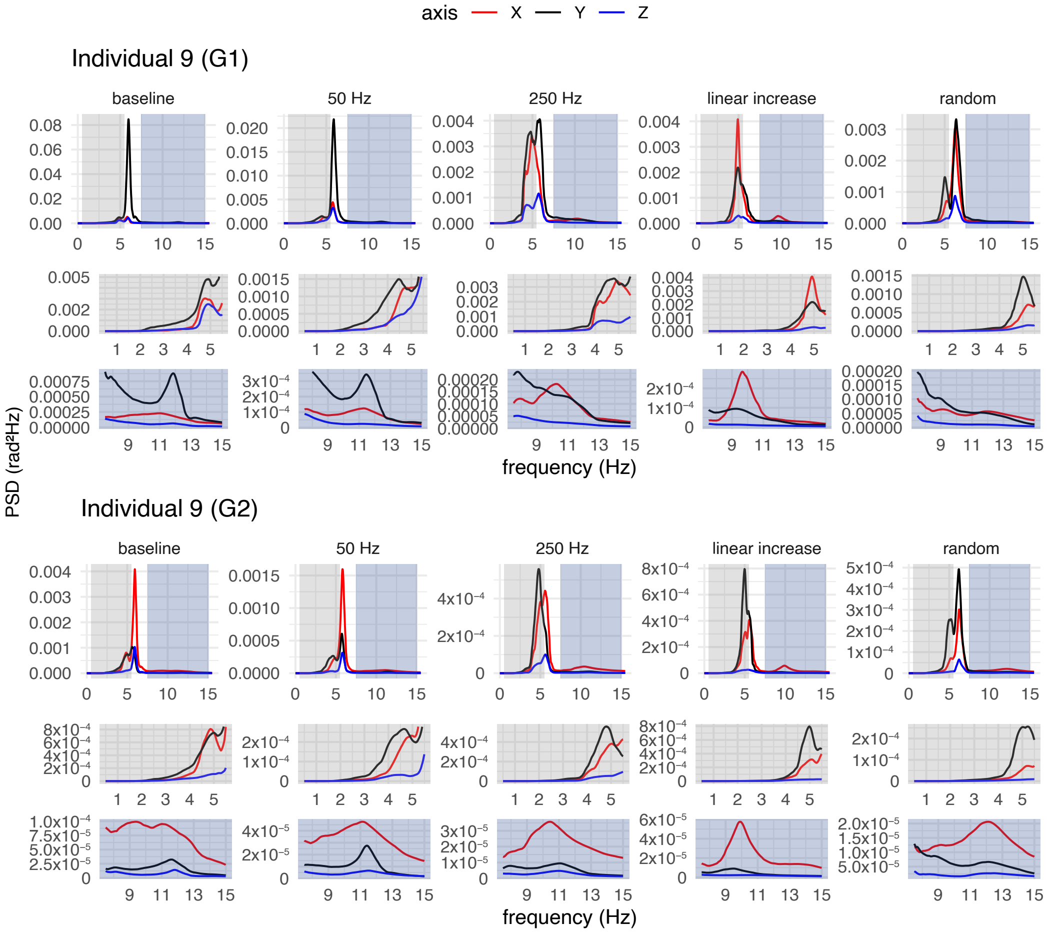

Figure S7. Power spectral densities of the angular velocity of the hand (G1) and forearm (G2) of **Individual 9** for each axis (X, Y and Z) during posture maintenance, without (baseline) and with (50 Hz, 250 Hz, linear and random increase) vibrotactile stimulation. The highlighted areas of the spectra (the gray and blue windows) depict the spectral behaviour for lower and higher frequency bands in relation to the peak frequency.

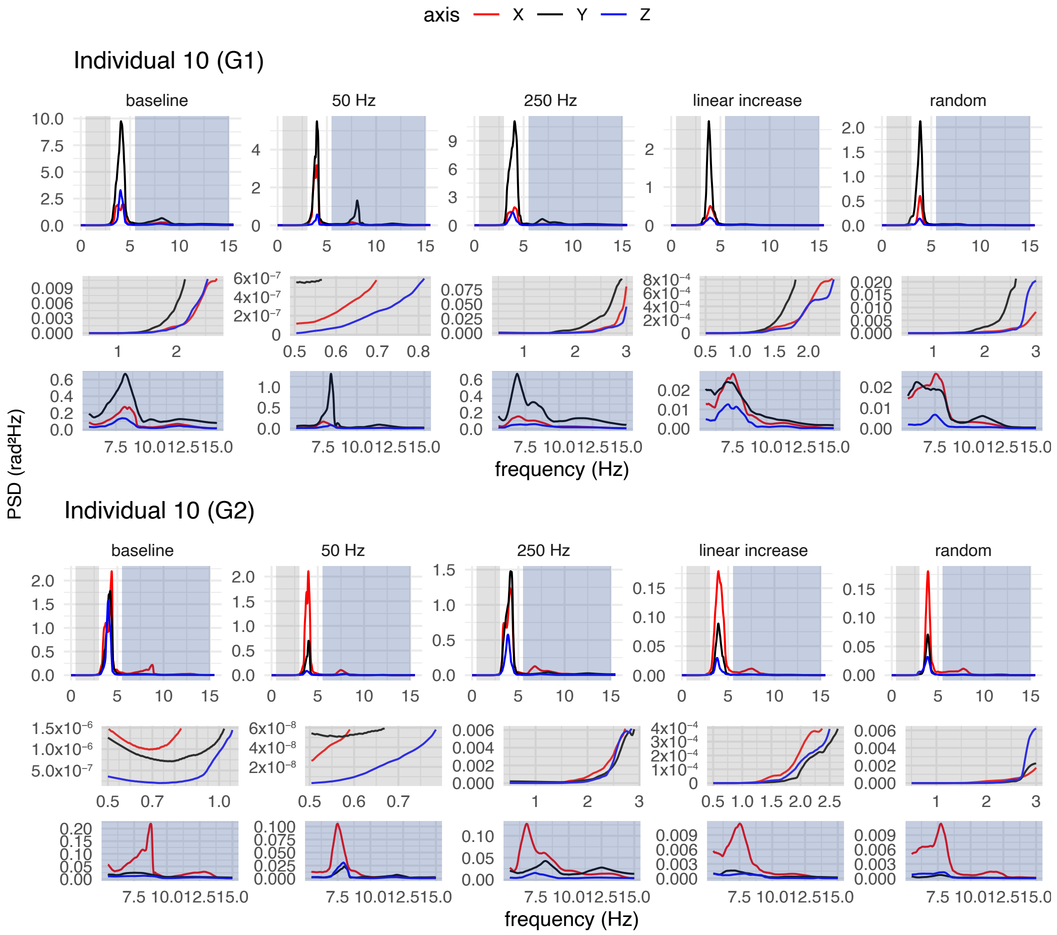

Figure S8. Power spectral densities of the angular velocity of the hand (G1) and forearm (G2) of **Individual 10** for each axis (X, Y and Z) during posture maintenance, without (baseline) and with (50 Hz, 250 Hz, linear and random increase) vibrotactile stimulation. The highlighted areas of the spectra (the gray and blue windows) depict the spectral behaviour for lower and higher frequency bands in relation to the peak frequency.

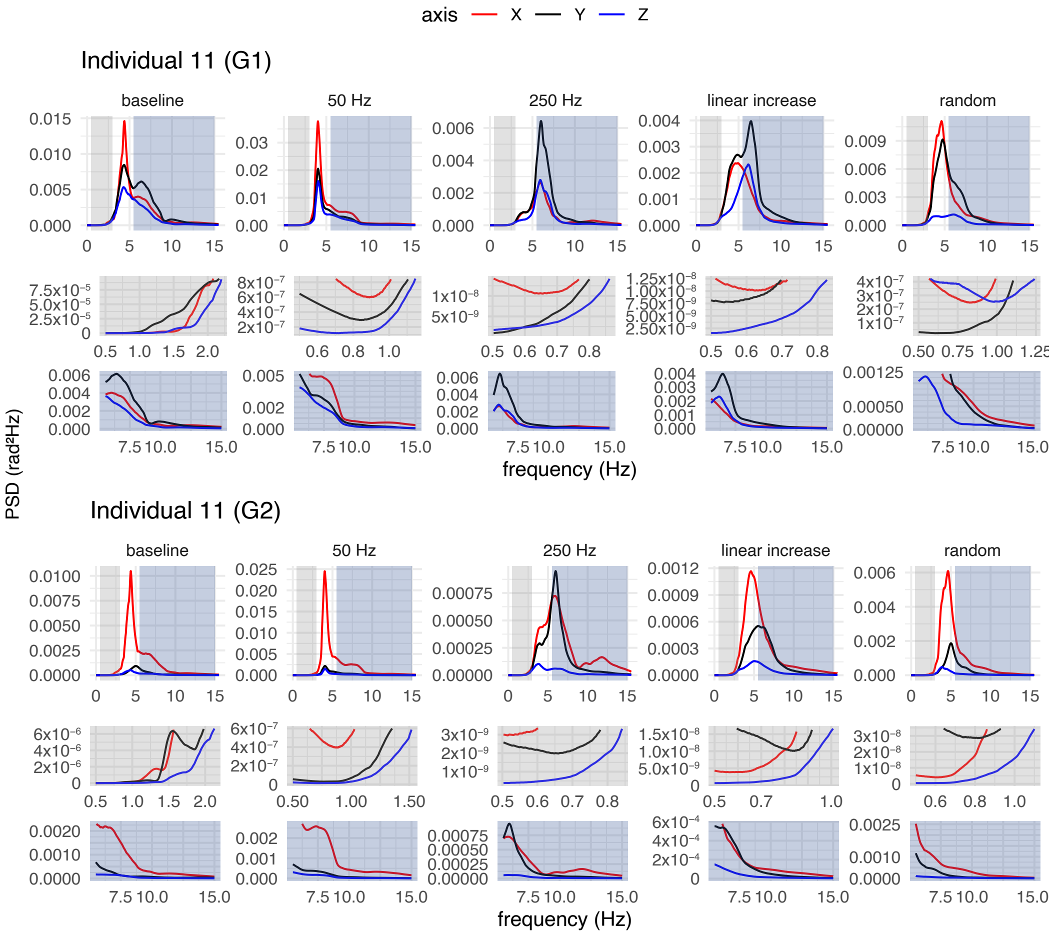

Figure S9. Power spectral densities of the angular velocity of the hand (G1) and forearm (G2) of **Individual 11** for each axis (X, Y and Z) during posture maintenance, without (baseline) and with (50 Hz, 250 Hz, linear and random increase) vibrotactile stimulation. The highlighted areas of the spectra (the gray and blue windows) depict the spectral behaviour for lower and higher frequency bands in relation to the peak frequency.

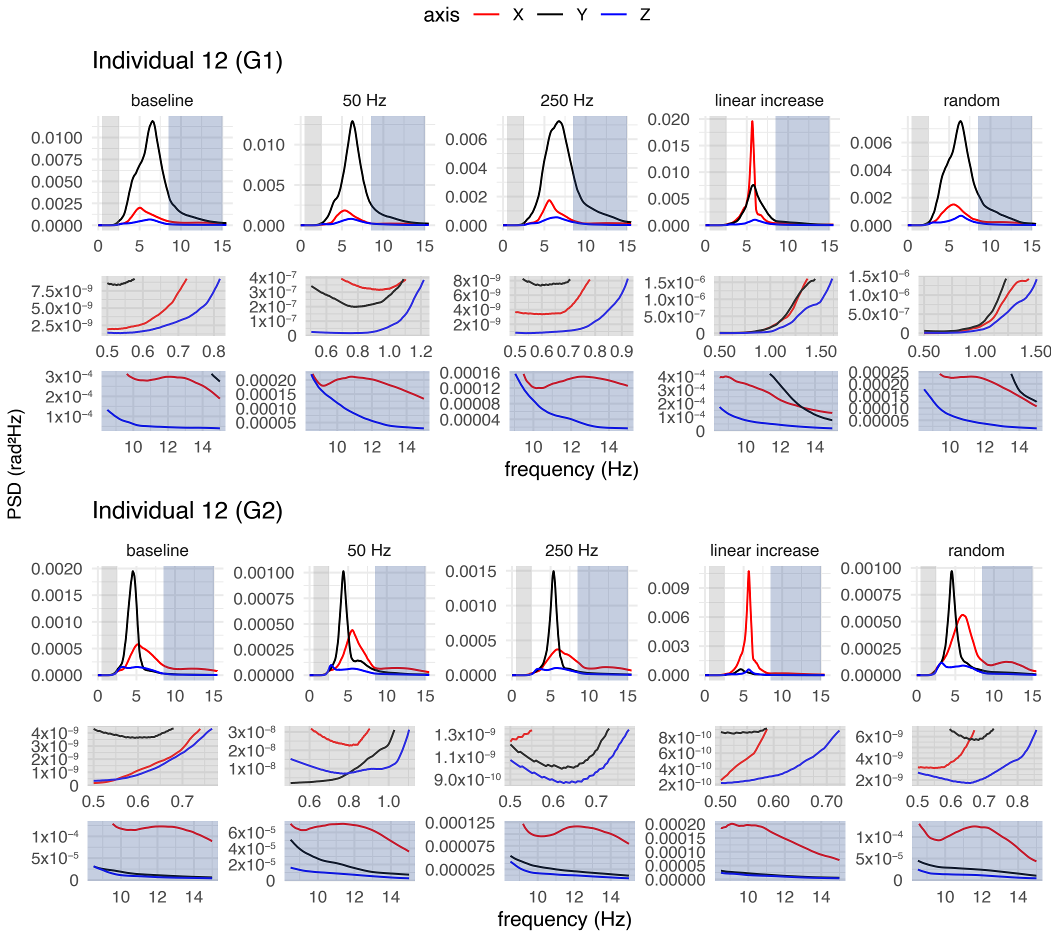

Figure S10. Power spectral densities of the angular velocity of the hand (G1) and forearm (G2) of **Individual 12** for each axis (X, Y and Z) during posture maintenance, without (baseline) and with (50 Hz, 250 Hz, linear and random increase) vibrotactile stimulation. The highlighted areas of the spectra (the gray and blue windows) depict the spectral behaviour for lower and higher frequency bands in relation to the peak frequency.

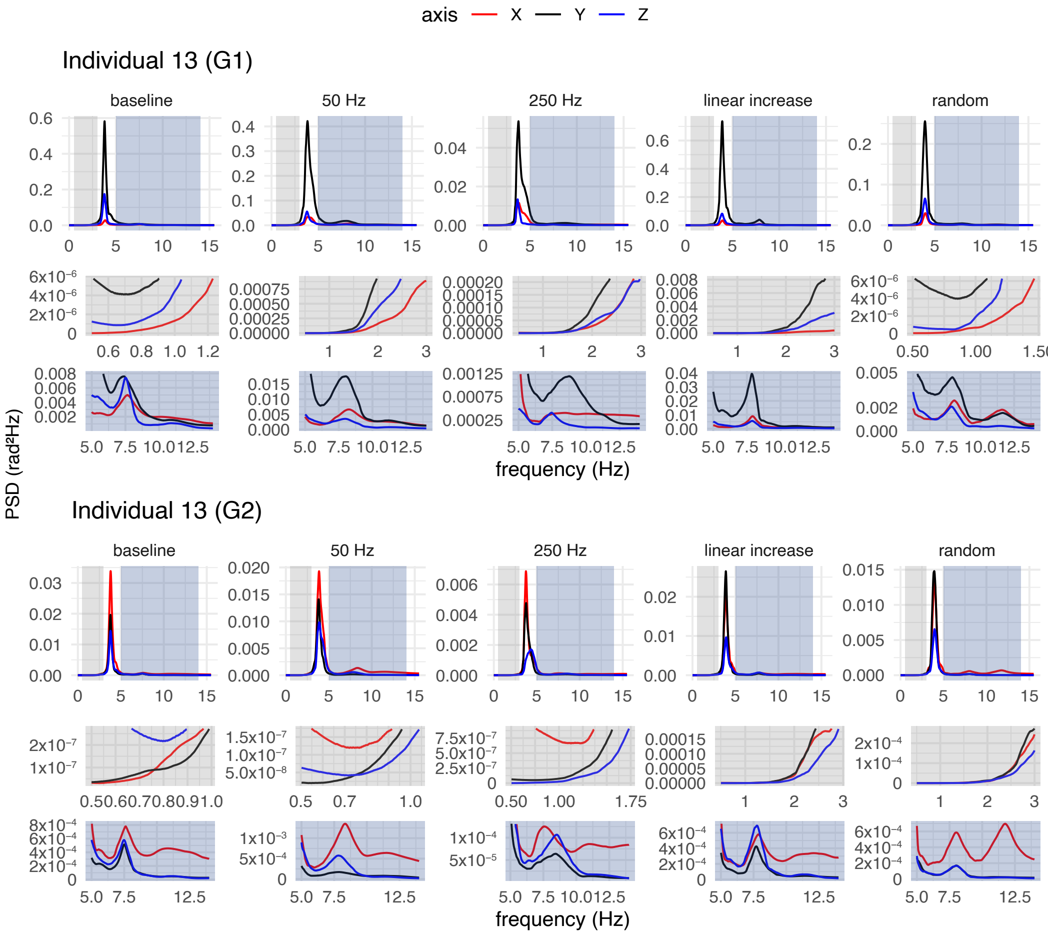

Figure S11. Power spectral densities of the angular velocity of the hand (G1) and forearm (G2) of **Individual 13** for each axis (X, Y and Z) during posture maintenance, without (baseline) and with (50 Hz, 250 Hz, linear and random increase) vibrotactile stimulation. The highlighted areas of the spectra (the gray and blue windows) depict the spectral behaviour for lower and higher frequency bands in relation to the peak frequency.

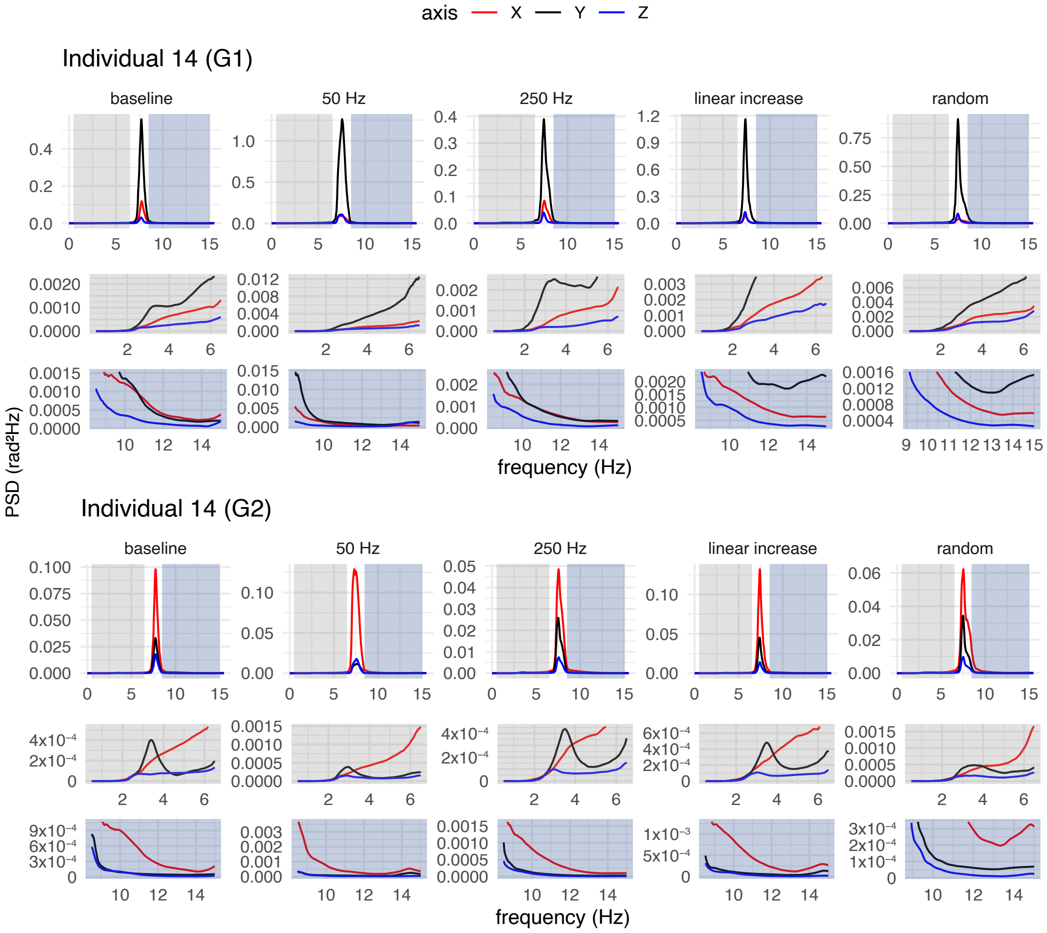

Figure S12. Power spectral densities of the angular velocity of the hand (G1) and forearm (G2) of **Individual 14** for each axis (X, Y and Z) during posture maintenance, without (baseline) and with (50 Hz, 250 Hz, linear and random increase) vibrotactile stimulation. The highlighted areas of the spectra (the gray and blue windows) depict the spectral behaviour for lower and higher frequency bands in relation to the peak frequency.

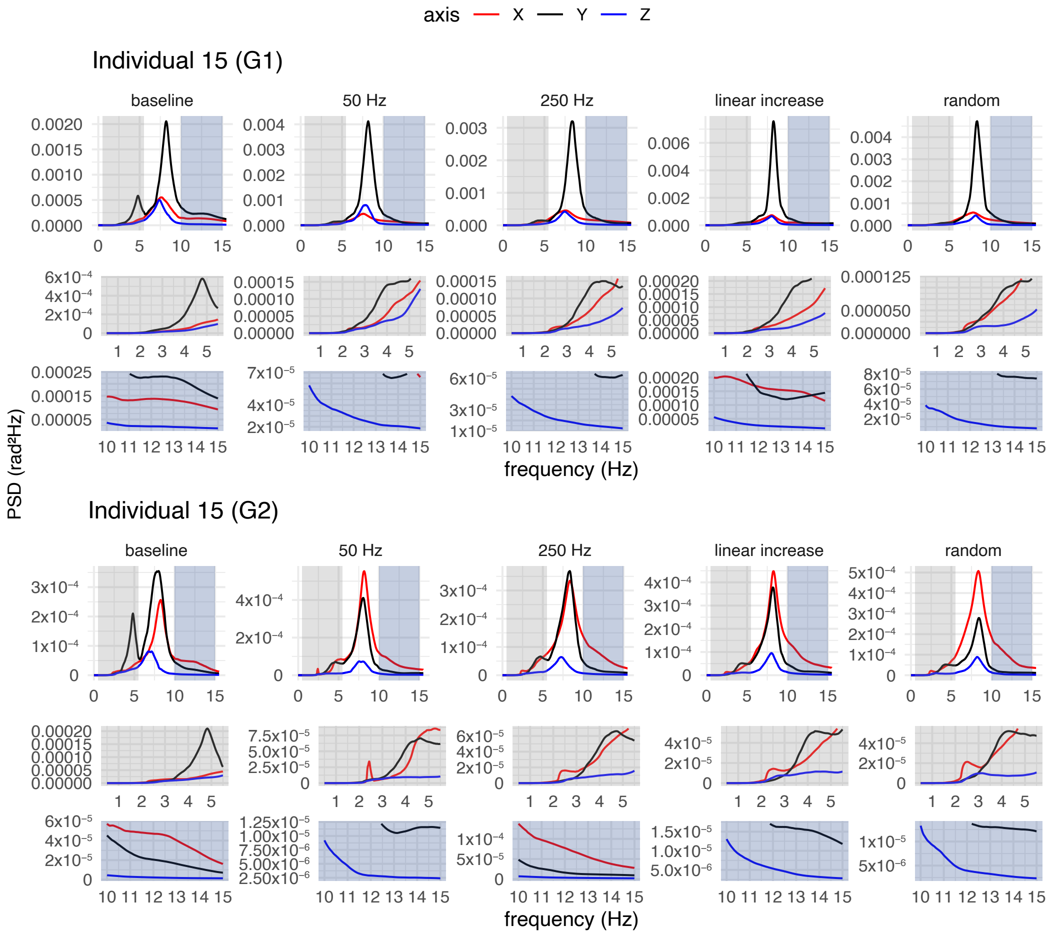

Figure S13. Power spectral densities of the angular velocity of the hand (G1) and forearm (G2) of **Individual 15** for each axis (X, Y and Z) during posture maintenance, without (baseline) and with (50 Hz, 250 Hz, linear and random increase) vibrotactile stimulation. The highlighted areas of the spectra (the gray and blue windows) depict the spectral behaviour for lower and higher frequency bands in relation to the peak frequency.

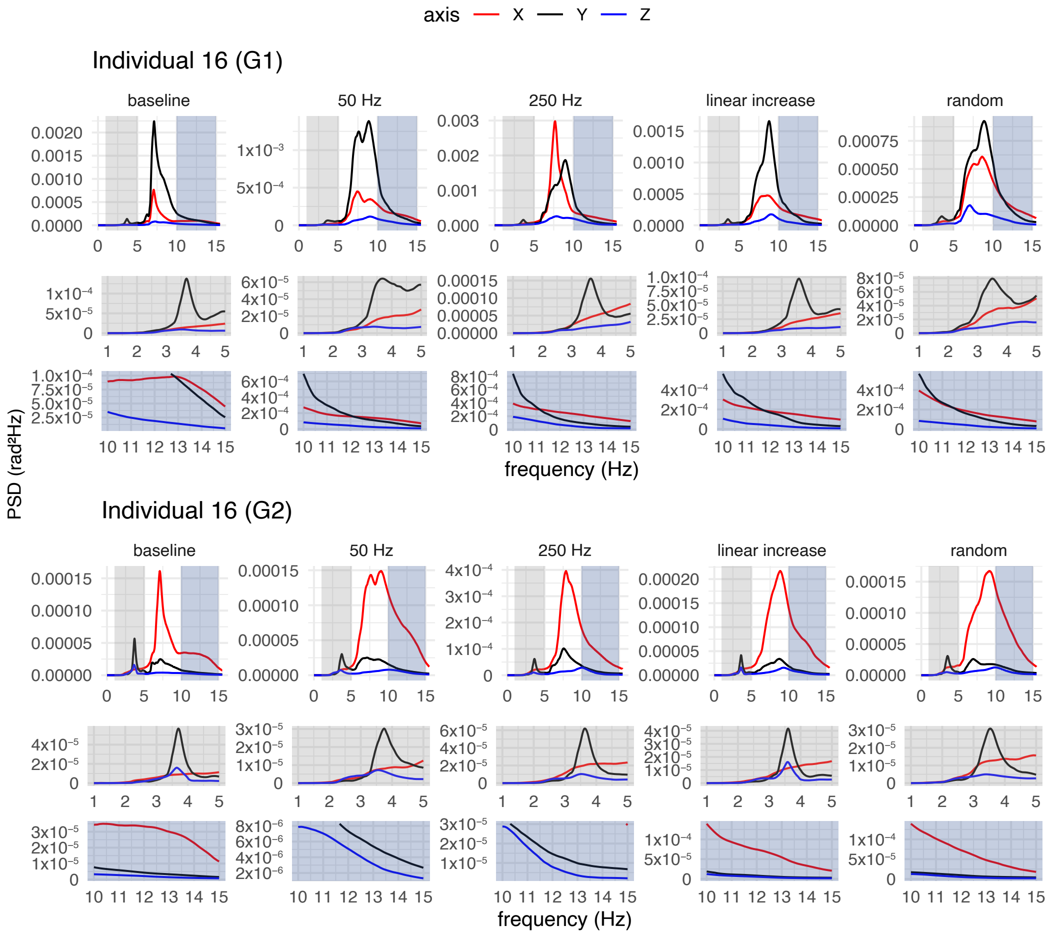

Figure S14. Power spectral densities of the angular velocity of the hand (G1) and forearm (G2) of **Individual 16** for each axis (X, Y and Z) during posture maintenance, without (baseline) and with (50 Hz, 250 Hz, linear and random increase) vibrotactile stimulation. The highlighted areas of the spectra (the gray and blue windows) depict the spectral behaviour for lower and higher frequency bands in relation to the peak frequency.

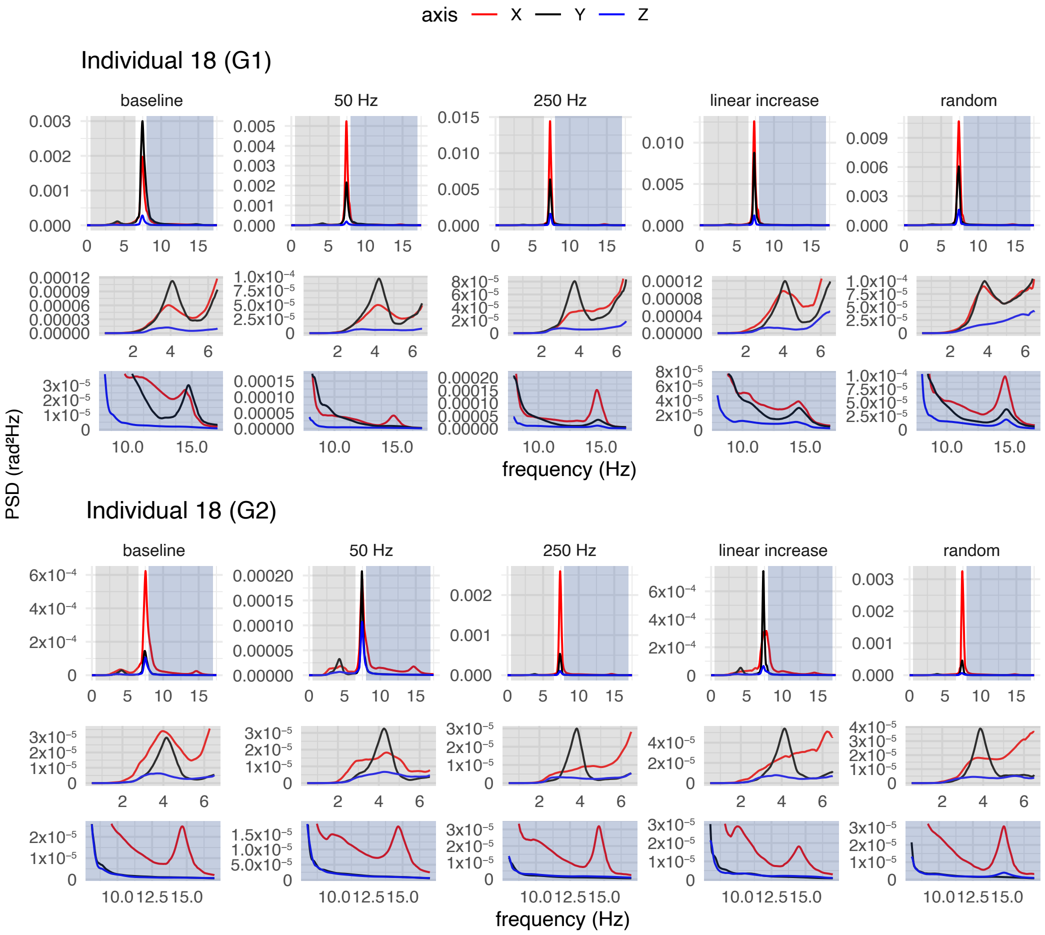

Figure S15. Power spectral densities of the angular velocity of the hand (G1) and forearm (G2) of **Individual 18** for each axis (X, Y and Z) during posture maintenance, without (baseline) and with (50 Hz, 250 Hz, linear and random increase) vibrotactile stimulation. The highlighted areas of the spectra (the gray and blue windows) depict the spectral behaviour for lower and higher frequency bands in relation to the peak frequency.
